# Responsive Neurostimulation of the Thalamus for the Treatment of Refractory Epilepsy

**DOI:** 10.3389/fnhum.2022.926337

**Published:** 2022-07-15

**Authors:** Jorge A. Roa, Marina Abramova, Madeline Fields, Maite La Vega-Talbott, Jiyeoun Yoo, Lara Marcuse, Steven Wolf, Patricia McGoldrick, Saadi Ghatan, Fedor Panov

**Affiliations:** ^1^Department of Neurosurgery, Icahn School of Medicine at Mount Sinai, New York, NY, United States; ^2^Department of Neurology, Icahn School of Medicine at Mount Sinai, New York, NY, United States; ^3^Department of Neurology, Boston Children's Health Physicians, New York Medical College, New York, NY, United States

**Keywords:** responsive neurostimulation (RNS), thalamus, refractory epilepsy, outcomes, complications

## Abstract

**Introduction:**

One-third of patients with epilepsy continue to have seizures despite antiepileptic medications. Some of these refractory patients may not be candidates for surgical resection primarily because the seizure onset zones (SOZs) involve both hemispheres or are located in eloquent areas. The NeuroPace Responsive Neurostimulation System (RNS) is a closed-loop device that uses programmable detection and stimulation to tailor therapy to a patient's individual neurophysiology. Here, we present our single-center experience with the use of RNS in thalamic nuclei to provide long-term seizure control in patients with refractory epilepsy.

**Methods:**

We performed a prospective single-center study of consecutive refractory epilepsy patients who underwent RNS system implantation in the anterior (ANT) and centromedian (CM) thalamic nuclei from September 2015 to December 2020. Patients were followed postoperatively to evaluate seizure freedom and complications.

**Results:**

Twenty-three patients underwent placement of 36 RNS thalamic leads (CM = 27 leads, ANT = 9 leads). Mean age at implant was 18.8 ± 11.2 years (range 7.8–62 years-old). Two patients (8.7%) developed infections: 1 improved with antibiotic treatments alone, and 1 required removal with eventual replacement of the system to recover the therapeutic benefit. Mean time from RNS implantation to last follow-up was 22.3 months. Based on overall reduction of seizure frequency, 2 patients (8.7%) had no- to <25% improvement, 6 patients (26.1%) had 25–49% improvement, 14 patients (60.9%) had 50–99% improvement, and 1 patient (4.3%) became seizure-free. All patients reported significant improvement in seizure duration and severity, and 17 patients (74%) reported improved post-ictal state. There was a trend for subjects with SOZs located in the temporal lobe to achieve better outcomes after thalamic RNS compared to those with extratemporal SOZs. Of note, seizure etiology was syndromic in 12 cases (52.2%), and 7 patients (30.4%) had undergone resection/disconnection surgery prior to thalamic RNS therapy.

**Conclusion:**

Thalamic RNS achieved ≥50% seizure control in ~65% of patients. Infections were the most common complication. This therapeutic modality may be particularly useful for patients affected by aggressive epilepsy syndromes since a young age, those whose seizure foci are located in the mesial temporal lobe, and those who have failed prior surgical interventions.

## Introduction

In 2016, there were 45.9 million patients with epilepsy worldwide (Collaborators, 2019). The prevalence of active epilepsy increases with age, and an estimated 70% of patients could be seizure free if properly diagnosed and treated. In the US, approximately 3.4 million people live with epilepsy (Zack and Kobau, 2017). Of those, about 10–40% never achieve adequate seizure control with medications, which results in significant detrimental impacts on quality of life and health (Kwan and Sander, 2004; Gesche et al., 2020). In some of these drug-refractory epilepsy (DRE) cases, patients may not be candidates for resective or ablative surgical treatments, primarily because the seizure onset zones (SOZs) involve both hemispheres or are located in eloquent areas. Brain stimulation, however, is a surgical therapy that holds great promise for reducing seizure burden in such patients.

The potential for using electrical stimulation to abort seizures has been demonstrated since early intraoperative explorations by Penfield and Jasper (Penfield and Jasper, 1954; Jasper et al., 1955). Similarly, the role of thalamic nuclei in generalized epilepsy circuitry has been extensively studied in both animal and human models. In 1949, Hunter and Jasper already demonstrated that seizures could be induced by electrical stimulation of the thalamus (Hunter and Jasper, 1949). Subsequently, Monnier et al. showed that medial thalamic stimulation could also exert two different desynchronizing effects on cortical electroencephalography (EEG); specifically, stimulation of the ascending reticular system predominantly activates various types of single cortical neurons, whereas stimulation of the intralaminary projecting system may inhibit the same single cortical units (Monnier et al., 1960). In 1987, Velasco et al. began exploring the centromedian (CM) nucleus of the thalamus as a target for neurostimulation for DRE with excellent results, including an improvement in psychological performance beyond that expected by reduction in seizure activity. This improvement was noticed when comparing baseline and 3-month evaluations of psychological performance through selected Beta R, Wechsler memory scale, visual discrimination, Minnesota Multiphasic Personality Inventory, and Zung's rated depression scale (Velasco et al., 1987). Later on, Valentin et al. performed a single-blind control trial by placing bilateral deep brain stimulation (DBS) electrodes in the CM of 11 patients with refractory generalized epilepsy or frontal lobe epilepsy, achieving >50% improvement in the first group (Valentin et al., 2013).

The Stimulation of the Anterior Nucleus (ANT) of the Thalamus for Epilepsy (SANTE) trial demonstrated the benefit of DBS of the ANT for treating secondary generalization of focal seizures after 2-year (Fisher et al., 2010) and 5-year (Salanova et al., 2015) follow-ups. In contrast to traditional DBS, responsive neurostimulation (RNS) is a closed-loop system that uses programmable detection and stimulation to tailor therapy to a patient's individual neurophysiology, and has demonstrated reductions in frequency and severity of focal seizures. As this device provides responsive stimulation directly to the seizure circuit when epileptiform activity is detected, the intent is to disrupt epileptiform activity before a full seizure develops (Morrell, 2011; Heck et al., 2014; Geller et al., 2017).

The NeuroPace RNS System (Mountain-view, CA, USA) is the only closed-loop neurostimulation device approved by the Food and Drug Administration (FDA) for use in patients with DRE (FDA, 2022). Our prior experience with RNS in 27 pediatric patients showed significant improvement in seizure frequency in all cases (Panov et al., 2020). Here, we present outcomes for our initial series of twenty-three consecutive adult and pediatric patients with DRE who underwent thalamic RNS in the ANT or CM at our center. Our results suggest that the thalamus is a valuable target for both detection and stimulation because of its critical role in early seizure propagation in well selected patients.

## Methods

### Patient Selection

Subjects had a consensus diagnosis of DRE, which included epilepsy patients who had failed to respond to two adequate trials of antiseizure medications alone or in combination, or who had not responded to prior interventions such as laser ablation, surgical resection, or VNS implantation. All patients underwent our extensive institutional protocol for seizure work-up which, in accordance with current published guidelines, includes volumetric computerized tomography (CT), magnetic resonance imaging (MRI), video-EEG, functional MRI, Wada testing, magnetoencephalography, and positron emission tomography as indicated (Ryvlin et al., 2021). Diagnostic intracranial EEG with either cortical grids/strips or stereo-EEG was performed to characterize SOZs, and a consensus was reached to rule out resective and ablative options. Following the recommendation of a multidisciplinary surgical epilepsy conference, patients underwent RNS implantation in the ANT or CM nuclei of the thalamus. No patients were excluded from the study or the analysis.

Subjects were studied prospectively via an institutional review board (IRB)-approved epilepsy surgery database, for which they provided consent for inclusion. IRB review was not used for implantation of RNS in the thalamus. The off-label use was to provide the best potential treatment option for reducing seizures in these patients, based on the expert opinion of the multidisciplinary epilepsy team.

### Implantation Technique

**Figure 1:**
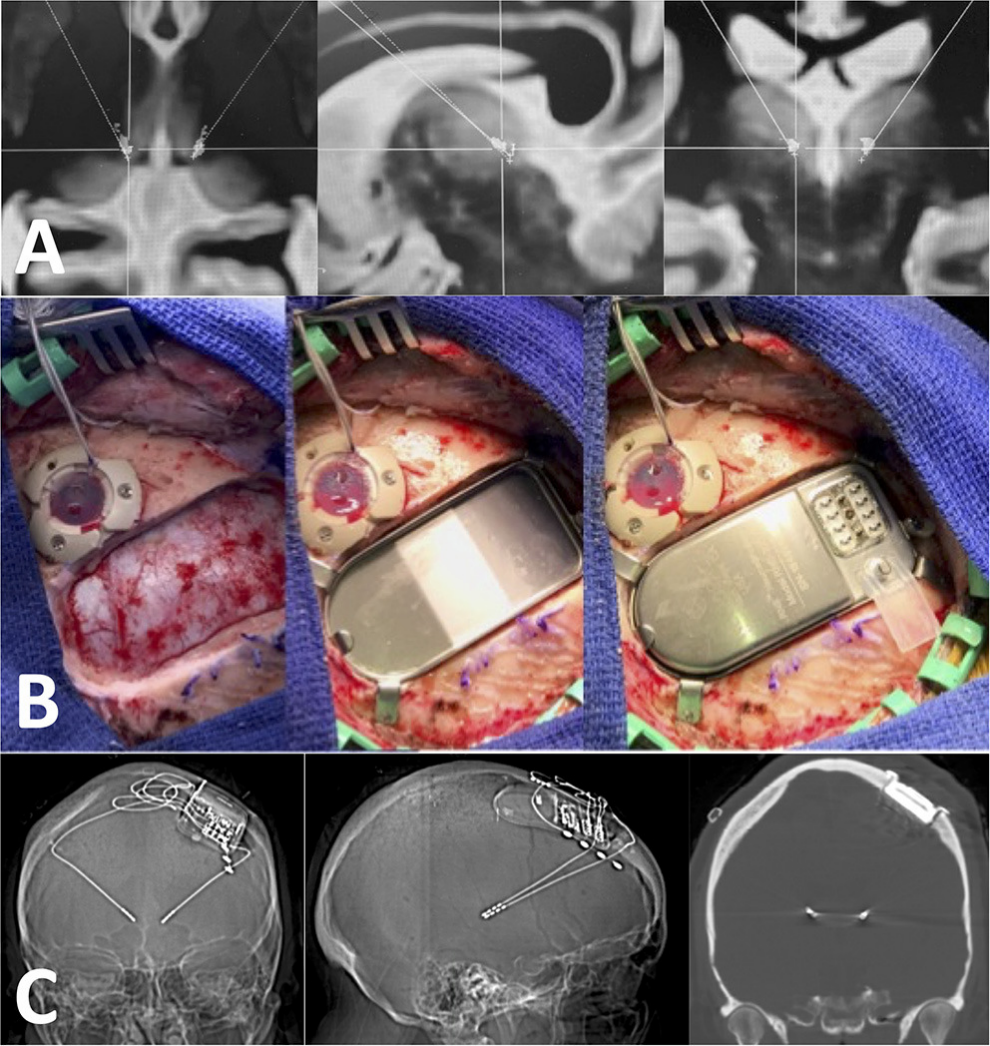
Technique used for RNS implantation. **(A)** Axial, sagittal, and coronal images showing bilateral target points in the CM. These targets are determined using T1 post-contrast, FGATIR and MPRAGE sequences in the ROSA software. **(B)** Intraoperative pictures showing the ferrule and the tray for the neurostimulator. Notice how the ferrule allows the neurostimulator to be “flushed” with the bone cortical surface at the craniotomy site. **(C)** Post-operative volumetric CTH (bone window) showing good placement in the CM with no tract hemorrhages.

Targeting for RNS device implantation in the ANT or CM was performed on 3T MRI volumetric sequences with gadolinium merged with FGATIR or MPRAGE sequences for direct target visualization using ROSA robot software (Zimmer Biomet, Warsaw, IN, USA). Implantations were planned considering the primary and alternative areas of detection and stimulation (Figure 1). Placement of the ferrule and the tray for the neurostimulator was based on skull thickness and ease of neurostimulator replacement or revision. Patients were positioned supine with head pin fixation using the Leksell base ring (Elekta). The head holder was firmly attached to the robot and coregistered by selecting bone fiducials localized on an intraoperative volumetric CT or O-arm–acquired volumetric scan (Medtronic) (Faraji et al., 2020). The intraoperative volumetric scan was fused with preoperative MRI images, and a satisfactory fiducial coregistration error (under 1 mm) was confirmed in all cases. Minimal head shaving was performed to accommodate the ferrule incision and the smaller depth electrode entry incisions. The head was prepped and draped in a sterile fashion, and cefazolin was administered in weight-based dosages intravenously within 1 h of the incision, unless a documented penicillin allergy prompted the use of vancomycin.

Depth electrodes were placed first to limit target error due to brain shift. When cortical strips were needed, we subsequently placed those utilizing loss of cerebrospinal fluid at the small dural opening at the ferrule site. Intraoperative electrocorticography was done in all cases. All patients had a volumetric head CT after extubation to rule out malposition and track hemorrhage. Accurate placement of all depth leads was confirmed by merging the postoperative volumetric CT to the preoperative targeting MRI. All patients were observed for the first night in either the pediatric or neurosurgical ICU, and the majority were discharged home the next day.

### RNS Programming

Prior to the initiation of stimulation, the RNS was set to record intracranial EEG without stimulation to characterize baseline thalamic electrophysiology. Electrophysiological data were extracted from the NeuroPace Patient Data Management System (PDMS). Follow-up appointments were scheduled at approximately 1- and 3-month intervals for programming adjustments and monitoring. The percentage of seizure vs. non-seizure stimulation was calculated from reviewed intracranial EEG recordings, and a tailored stimulation protocol was applied for each patient as needed.

### Outcome Variables

Seizure outcomes (frequency, duration, severity, post-ictal state, and reduction in antiseizure medications) were prospectively assessed with a minimum follow-up of 6 months after RNS implantation. All patients were carefully followed up by the epileptologist. Outcomes were quantified based on a diary recording the number, severity, and duration of seizure events throughout the week. Those records are carefully reviewed by the team during the follow-up appointments, and compared with the PDMS electrophysiological data. Finally, the patient and/or guardian provided an overall impression of how much improvement was achieved. Based on the relative reduction in seizure frequency, patients were stratified into quartiles as follows: group I, seizure free; group II, 50–99%; group III, 25–49%; and group IV, 0–24% (including no improvement).

## Results

### Demographics

A total of twenty-three patients underwent placement of RNS in thalamic nuclei over this study period. Table 1 provides an overall description of the patients' demographics and seizure outcomes. There was a slightly higher prevalence of males (14 patients, 60.9%). A significant number of subjects were in the pediatric population: the mean age at implant was 18.8 ± 11.2 years (range 7.8–62 years-old).

**Table 1:** Baseline demographics and seizure outcomes of the sample.

**No**.	**Age at implant (years)/Gender**	**Years with epilepsy/etiology**	**Seizure classification**	**Target**	**Prior procedure**	**Follow-up (months)**	**Seizure outcome**
1	28/F	19/Idiopathic	Generalized temporal plus	Lt SMA/Lt ANT	VNS	15	Seizure-free
2	11/F	11/ FCD type IA	Generalized multilobar	Rt ANT/Lt Hippocampus	Rt temporal lobectomy + frontal disconnection	38	75–99%
3	12/M	10/Dup15q	Generalized bilat temporal	Lt CM/Rt CM	No	12	75–99%
4	9/M	9/ Idiopathic	Complex partial temporal plus	Lt CM/Rt Frontal	No	6	75–99%
5	17/M	7/Idiopathic	Generalized bilat temporal	Lt CM/Rt Hippocampus	No	11	75–99%
6	24/F	8/Idiopathic	Generalized bilat temporal	Lt CM/Rt CM	No	8	75–99%
7	17/M	4/Idiopathic	Generalized bilat temporal	Lt CM/Rt CM	No	6	75–99%
8	14/M	14/ Syndromic	Generalized multilobar	Lt ANT/Rt Temporal	Rt frontal lobectomy + callosotomy + VNS	54	50–74%
9	12/F	11/TB meningitis	Generalized multilobar	Lt CM/Lt Frontal	Rt hemispherectomy + frontal lobe disconnection + callosotomy	29	50–74%
10	10/F	10/LGS	Generalized temporal plus	Rt Hippocampus/Lt CM	Callosotomy	30	50–74%
11	21/M	8/Syndromic	Generalized temporal plus	Lt Frontal/Rt CM	No	32	75–99%
12	8/M	6/ Microcephaly	Generalized temporal plus	Lt CM/Rt CM	Rt frontal disconnection + Rt temporal lobectomy Amygdalohippocampectomy	18	50–74%
13	17/M	16/LGS	Generalized bilat temporal	Lt CM/Rt CM	Callosotomy	21	50–74%
14	15/F	11/LGS	Complex partial temporal plus	Lt CM/Rt Frontal	No	18	25–49%
15	31/M	29/DDMS	Generalized bilat temporal	Lt CM/Rt CM	VNS	32	No change/ <25%
16	25/M	11/GCT	Generalized multilobar	Lt CM/Rt CM	No	13	25–49%
17	62/M	3/Viral meningitis	Generalized bilat temporal	Lt CM/Rt CM	No	10	25–49%
18	10/F	2/DCX mutation	Generalized bilat temporal	Lt CM/Rt CM	No	27	25–49%
19	17/M	9/Idiopathic	Generalized temporal plus	Lt CM/Rt CM	Callosotomy	33	75–99%
20	20/M	7/LGS	Complex partial bilat temporal	Lt ANT/Rt ANT	No	30	50–74%
21	14/F	8/PMS	Generalized multilobar	Lt ANT/Rt ANT	No	23	No change/ <25%
22	18/M	13/Idiopathic	Generalized temporal plus	Lt SMA/Rt CM	No	16	25–49%
23	21/F	14/Idiopathic	Generalized bilat temporal	Lt ANT/Rt ANT	No	33	25–49%

*ANT, anterior thalamic nucleus; Bilat, bilateral; CM, centromedian thalamic nucleus; DCX, doublecortin; DDMS, Dyke-Davidoff-Masson syndrome; F, female; FCD, focal cortical dysplasia; GCT, germ cell tumor; LGS, Lennox-Gastaut syndrome; Lt, left, M, male; PMS, Phelan-McDermid syndrome; Rt, right; SMA, supplementary motor area; TB, tuberculosis, VNS, vagal nerve stimulator*.

### Baseline Characteristics

Mean age of seizure onset was 8.5 ± 12.1 years (range 2 months−59 years-old). The mean number of years suffering from epilepsy prior to RNS implantation was 10.4 ± 5.7 years (range 2.1–29 years). The etiology for epilepsy was syndromic in most cases (12, 52.2%), followed by idiopathic (8, 34.8%), infectious/meningitis (2, 8.7%) and tumor (1, 4.3%). The most common type of seizure was generalized tonic-clonic (21, 91.3%), with a minority of patients suffering focal seizures with impaired awareness (2, 8.7%). The SOZ was characterized as bilateral temporal in 10 (43.5%) cases, temporal plus in 8 (34.8%), and multilobar in 5 (21.7%) cases. Prior to RNS implantation, 7 patients (30.4%) had undergone resection/disconnection surgery, and 3 patients (13%) had failed VNS.

### RNS Placement

All patients underwent placement of bilateral leads. A total of 36 thalamic leads (CM = 27 leads, ANT = 9 leads) were implanted. A total of 13 patients (56.5%) had thalamic leads placed bilaterally (CM = 10 patients/20 leads, ANT = 3 patients/6 leads), whereas the remaining 10 patients (43.5%) had one thalamic lead on one side (CM = 7 patients/7 leads, ANT = 3 patients/3 leads) and another depth lead (hippocampus = 3 patients/3 leads) or cortical contralateral lead (frontal/supplementary motor area = 6 patients/6 leads, temporal = 1 patient/1 lead). No intraoperative complications occurred.

### Post-operative Complications

Two patients (8.7%) developed infections: 1 (4.3%) patient improved with antibiotic treatments alone, and 1 required removal of the system, post removal antibiotic course with eventual replacement of the system to recover the therapeutic benefit. No post-operative track-hemorrhages were documented in this cohort. No cases of clinical stroke, device migration or malfunction were seen.

### Seizure Outcomes During Follow-Up

Mean time from RNS implantation to last follow-up was 22.4 months (6 months−4.5 years). Based on overall reduction of seizure frequency, 2 patients (8.7%) had no- to <25% improvement, 6 patients (26.1%) had 25–49% improvement, 14 patients (60.9%) had 50–99% improvement, and 1 patient (4.3%) became seizure-free. A paired *T*-test comparing seizure frequency at baseline and at last follow-up demonstrated a statistically significant effect of thalamic RNS (*p* = 0.042). All patients reported significant improvement in seizure duration and severity, and 17 patients (74%) reported improved post-ictal state. Fifteen patients (65.2%) did not experience any reductions in their AEDs regimen. No patients had increased need of antiseizure medications after thalamic RNS. Medication decrease was performed progressively, after reaching a steady state in RNS settings, thus assuring no confounders in outcomes. Additionally, 19/23 (82.6%) patients with 1-year or longer follow-up underwent a formal neuropsychiatric assessment and had no adverse effects reported.

An important observed trend in our cohort was the fact that subjects with SOZs located in the temporal lobe achieved relatively better outcomes after thalamic RNS compared to those with seizures that were multifocal in origin. The patient who achieved seizure freedom was a 28-year-old female who had been suffering from DRE for 19 years which had not responded to VNS therapy, were idiopathic in origin, with a SOZ localized to the temporal lobe with rapid spread; she had a left SMA cortical strip and a left ANT depth implant. In contrast, of the two patients who did not experience significant improvement in our cohort, one was a 14-year-old female with a bilateral ANT implant, who had been suffering from refractory generalized seizures associated with Phelan-McDermid syndrome for 8 years, with work up revealing a very diffuse multilobar SOZ. The other patient, a 31-year-old male with 29 years of refractory epilepsy secondary to Dyke-Davidoff-Masson syndrome, received a bilateral CM implant as his SOZ was also multilobar, and he had previously failed VNS therapy.

## Discussion

To our knowledge, our study represents the largest published cohort of patients with DRE who underwent thalamic RNS with seizure outcomes up to 4.5 years. Our results suggest that thalamic RNS is an effective treatment modality. In fact, around one-third of patients in our sample had not only failed two trials of anti-seizure medications, but also had failed prior attempts of surgical resection/disconnection procedures or VNS therapy. Moreover, in over half of the patients, their refractory seizures were associated with aggressive epileptic syndromes. This is a particularly difficult-to-treat population with no options remaining. Thalamic RNS may be able to reduce seizure frequency and severity in this group, resulting in readily appreciable quality-of-life improvement.

### RNS of the CM

The thalamus is the primary relay center of the brain, sending all sensory information besides olfaction to the cerebral cortex, where it is further processed (Jones, 2002a). Thalamocortical interneurons receive sensory or motor information from the body and signal distinct thalamic nuclei to relay selected information via relay neurons in the thalamocortical radiations to the cerebral cortex (Jones, 2002b). Thus, it is not surprising that neurostimulation of thalamic nuclei exerts substantial regulatory effects in the threshold potentials of many surrounding white matter tracts, and has promising potential in the treatment of DRE.

Most thalamic electrodes in this study were implanted in the CM. Previous small case series using the CM as a target for RNS have shown promising results. Kokkinos et al. reported a substantial decrease of daily seizures from a mean of 60 to ≤ 10 in a patient diagnosed with eyelid myoclonia with absences after bilateral CM RNS (Kokkinos et al., 2020). Welch et al. also described a 16-year-old male with drug-resistant primarily generalized epilepsy with absence seizures, who achieved a 75% reduction in seizure frequency following bilateral CM RNS (Welch et al., 2021). Recently, Sisterson et al. applied bilateral CM RNS to 4 patients with refractory epilepsy and achieved a robust and durable reduction in seizure frequency and severity in all patients after 2 years (Sisterson et al., 2022). However, only one of those patients had been diagnosed with a syndromic etiology (juvenile myoclonic epilepsy). In summary, CM neurostimulation has been applied in adult patients for the treatment of refractory regional neocortical epilepsy, (Burdette et al., 2020) generalized epilepsy (Kokkinos et al., 2020), LGS (Velasco et al., 2006; Kwon et al., 2020), and drug-resistant focal onset-seizures (Nair et al., 2020). Our study further expands the potential therapeutic benefit of CM RNS by including young patients with aggressive epileptic syndromes.

The mechanism by which neurostimulation of the CM aborts seizures is not completely understood. The CM receives converging input from the cortex, basal ganglia, and brainstem and participates in cognition (attention and arousal) and sensorimotor coordination (Ilyas et al., 2019). Thalamocortical feedback loops regulate cortical input during wakefulness to maintain attention and awareness, and its suppression may explain the initial loss of awareness associated with absence and generalized seizures (Kostopoulos, 2001). Neurostimulation of the CM may improve awareness and prevent spreading seizure discharges by disrupting the low-frequency ictal thalamocortical recruitment (Gummadavelli et al., 2015).

Repeated electrical stimulation of the brain in animal models of epilepsy can produce kindling (Goddard et al., 1969). As the thalamus displays a crucial role in neuropsychological functioning and performance, its repeated neurostimulation could potentially induce adverse effects. In our small cohort, we did not encounter any neuropsychological adverse events after thalamic RNS. This finding may be explained by the fact that RNS provides neurostimulation only in response to epileptogenic changes in a tailored fashion (and not continuously at a set pace as with DBS), thus reducing the likelihood of thalamic kindling-related adverse effects.

### RNS of the ANT

On the other hand, the ANT has been explored as a potential therapeutic target in epilepsy for a longer time. Before neurostimulation, stereotactic lesions of the ANT reportedly improved seizure control in human subjects (Mullan et al., 1967). In 1984, the initial experiments by Cooper et al. demonstrated successful regulation of evoked metabolic responses in the limbic system produced by stimulation of the ANT (Cooper et al., 1984). These studies were the first hint to suggest that the antiseizure potential of the ANT may be largely attributed to its important regulatory function in the limbic-striate system.

In 1988, Sussman et al. reported a series of five patients with intractable epilepsy (4 with complex partial seizures of temporal lobe origin, and one with secondary generalized tonic-clonic seizures). Three of the 5 patients showed improvement after ANT neurostimulation (Sussman, 1988). In 1997, a pentylenetetrazol-induced seizure model in rats showed that high-frequency electrical stimulation of the ANT decreases seizures by increasing the threshold required to trigger uncontrolled discharges in the surrounding neuronal circuits (Mirski et al., 1997). As part of the mentioned SANTE trial, there was a 56% median percent reduction in seizure frequency, and 54% of patients had a seizure reduction of at least 50% after 2 years of DBS therapy targeting the ANT (Fisher et al., 2010). In the 5-year follow-up, 16% of subjects were seizure-free for at least 6 months (Salanova et al., 2015). The relatively higher benefit of thalamic RNS that we observed in patients with temporal SOZs may be partially explained by the integrative connections of the mesial temporal lobe with the ANT, parahippocampus, mammillary bodies and cingulate gyrus as part of the limbic circuit of Papez (1937). We predict that a larger cohort of DRE patients with thalamic RNS and with a longer follow-up will achieve similar results than those reported using DBS in the thalamus.

### Future Research

Given the variable response rates to stimulation of different thalamic nuclei, and their unique functional connectivity, the precise anatomic location target stimulation should be an important clinical consideration (Alcala-Zermeno et al., 2021). It may still be possible to get some indirect stimulation of the ANT by a depth electrode placed in the CM and vice versa. Neural plasticity induced by programmable closed-loop stimulation seems to play an important role in this process, as progressively better outcomes are achieved over months to years after RNS implantation (Warren et al., 2020). Target selection based on the seizure circuitry needs to be further elucidated. Seeding the thalamic target using DTI tractography and checking connectivity to the putative seizure focus is a valuable method in dire need of standardization. Programming of the thalamic RNS is an uncharted frontier and a challenge due to the degrees of freedom provided by detection and stimulation variations, with many likely advances over the next decade (Rossi et al., 2010; Garibay-Pulido et al., 2019). Combining data across centers with significant volumes will be key in providing answers to the above. A phase 3 clinical trial to study thalamic RNS as an adjunctive therapy for treating refractory generalized epilepsy in individuals ≥12 years old is planned to begin enrolling in 2022 (ClinicalTrials.gov Identifier: NCT05147571).

### Limitations

This is a small case series which limits the generalizability of any findings reported. Over half of the cohort included pediatric patients affected by epileptic syndromes; thus, our results may not be extrapolated to patients in other age groups with DRE of diverse etiology. Although the unique patient physiology present challenges for optimizing detection and stimulation, ANT and CM RNS can rapidly detect and abort ictal events in patients with DRE. Results from RNS in both ANT and CM thalamic targets have been combined in our results. With our current small sample, it is difficult to reach meaningful conclusions in terms of which thalamic target has a better outcome. It is important to consider that thalamic neurostimulation can induce subtle decrements across cognitive domains, and particularly in verbal memory (Dhima et al., 2021). Unfortunately, no objective cognitive measurements or tests were collected as part of our study. Without the evaluation of psychological and cognitive effects in the long-term after thalamic RNS, our preliminary results may not be generalized among different patient's groups. Moreover, our assessment of seizure frequency was based on a patient-dependent metric (diary method). As both subjects and caregivers were not blinded to study participation, their records on seizure frequency are vulnerable to Hawthorne effect which could potentially bias our results.

## Conclusion

Thalamic RNS achieved ≥50% seizure control in ~65% of patients. Infections were the most common complication. This therapeutic modality may be particularly useful for patients affected by aggressive epilepsy syndromes since a young age, and those who have failed prior surgical interventions. The long-term psychological and cognitive effects of thalamic RNS remain to be determined. Optimal detection and stimulation locations and parameters are an active area of investigation.

## Data Availability Statement

The raw data supporting the conclusions of this article will be made available by the authors, without undue reservation.

## Ethics Statement

The studies involving human participants were reviewed and approved by Institutional Review Board (IRB) Icahn School of Medicine at the Mount Sinai Hospital. Written informed consent to participate in this study was provided by the participants' legal guardian/next of kin.

## Author Contributions

JR and FP: study conception and design. All authors: data collection. JR, SG, and FP: analysis and interpretation of results. JR, MA, and FP: draft manuscript preparation. All authors reviewed the results and approved the final version of the manuscript.

## Funding

SW has received clinical or research support from NeuroPace for the study described.

## Conflict of Interest

FP and SG are consultants for NeuroPace. The remaining authors declare that the research was conducted in the absence of any commercial or financial relationships that could be construed as a potential conflict of interest.

## Publisher's Note

All claims expressed in this article are solely those of the authors and do not necessarily represent those of their affiliated organizations, or those of the publisher, the editors and the reviewers. Any product that may be evaluated in this article, or claim that may be made by its manufacturer, is not guaranteed or endorsed by the publisher.
